# Serotonin syndrome treated with cyproheptadine using NPi from a digital pupillometer as a therapeutic indicator: A case report

**DOI:** 10.1097/MD.0000000000037852

**Published:** 2024-04-12

**Authors:** Kazuki Sugaya, Tomotaka Misawa, Makoto Onodera, Ken Iseki

**Affiliations:** a Department of Emergency Medicine, Fukushima Medical University School of Medicine, Fukushima City, Fukushima, Japan; b Department of Regional Emergency Medicine, Fukushima Medical University, Fukushima City, Fukushima, Japan.

**Keywords:** automated pupillometer, case report, cyproheptadine, intentional autointoxication, overdose

## Abstract

**Rationale:**

Serotonin syndrome is a potentially life-threatening condition resulting from the use of antidepressants, their interactions with other serotonergic medications, or poisoning. It presents with a triad of psychiatric, dysautonomic, and neurological symptoms and is sometimes fatal. While cyproheptadine is a specific treatment option, the optimal duration of its administration remains unclear. The purpose of this report is to quantitatively assess the endpoints of serotonin syndrome treatment. Based on the hypothesis that neurological pupil index (NPi) on a digital pupil recorder would correlate with the severity of the serotonin syndrome, we administered cyproheptadine using NPi as an indicator.

**Patient concerns:**

A patient with a history of depression was brought to our hospital after he overdosed on 251 tablets of serotonin and noradrenaline reuptake inhibitors.

**Diagnoses:**

On day 3, the patient was diagnosed with serotonin syndrome.

**Interventions:**

Cyproheptadine syrup was administered at 4 mg every 4 hours. The NPi of the automated pupillometer was simultaneously measured. On day 5, the NPi exceeded 3.0 cyproheptadine was discontinued.

**Outcomes:**

The patient was discharged on day 7.

**Lessons:**

The lack of considerable improvement during the treatment period suggests that the patient may have improved on his own. In this case, the relationship between NPi and the severity of serotonin syndrome could not be determined.

## 1. Introduction

Serotonin syndrome results from the use of antidepressant medications, their interactions, or intentional autointoxication.^[1]^ The Hunter Criteria defines the symptoms as follows: spontaneous clonus, induced clonus and excitation or sweating, ocular clonus and excitation or sweating, tremor and hyperreflexia, and ocular clonus or induced clonus in addition to hypertonia and a temperature higher than 38°C.^[2]^ Although mild cases often spontaneously resolve with discontinuation of the causative drug, severe cases are sometimes fatal.^[1,3]^ Cyproheptadine is recommended to treat severe cases.^[4]^ However, the optimal duration of treatment is unknown, and no surrogate markers have been identified to indicate the end of treatment for serotonin syndrome. Because the determination of improvement in physical findings is likely to vary from examiner to examiner, we used the neurological pupil index (NPi) of the pupils as an indicator for administration of cyproheptadine, based on the hypothesis that the NPi on digital pupillometers correlates with the severity of the serotonin syndrome. To our knowledge, this is the first paper to evaluate serotonin syndrome using NPi. Informed consent was obtained from patients for publication of this case report.

## 2. Case presentation

The patient was a 30-year-old man with depression. Owing to work-related troubles, he overdosed himself on approximately 200 tablets of 30mg mirtazapine, 23 tablets of 75 mg venlafaxine, and 28 tablets of 20 mg lurasidone. He was brought to our hospital after approximately 2 hours had passed and was admitted to the emergency department. Gastric lavage was not performed at the discretion of the emergency room physician.

When the patient arrived at our hospital, he scored 10 on Glasgow Coma Scale (E3V2M5), with body temperature, 37.3°C; blood pressure, 145/112 mm Hg; heart rate, 125 beats/min; respiratory rate, 26 breaths/minute; and oxygen saturation, 95% at rest in room temperature air. He was unable to communicate, his pupils were dilated to 6 mm/6 mm, and he had occasional upper-extremity tremors with hyperreflexia tendon reflexes. Blood tests and whole-body computed tomography (CT) showed no notable abnormalities. He did not admit the use of illegal drugs, and nothing was detected in a urine drug test.

After hospitalization, he developed psychiatric symptoms (agitation, hallucinations, and delusions). On day 2, the patient presented with autonomic (hypertension, tachycardia, and fever) and neurological (tremor and clonus) symptoms. The clonus was particularly prominent in the lower extremities.

**Figure 1.**
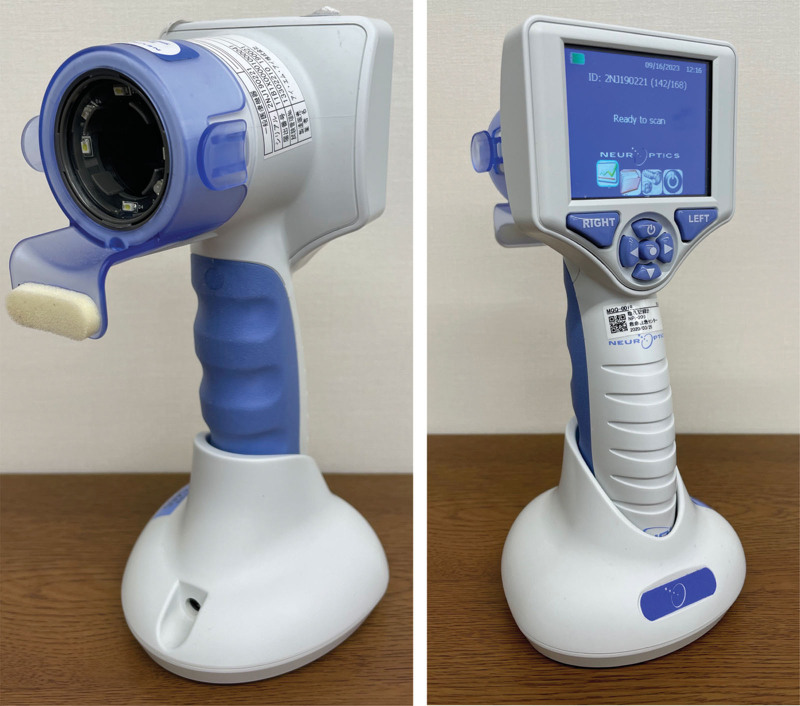
Digital pupillometer.

**Figure 2.**
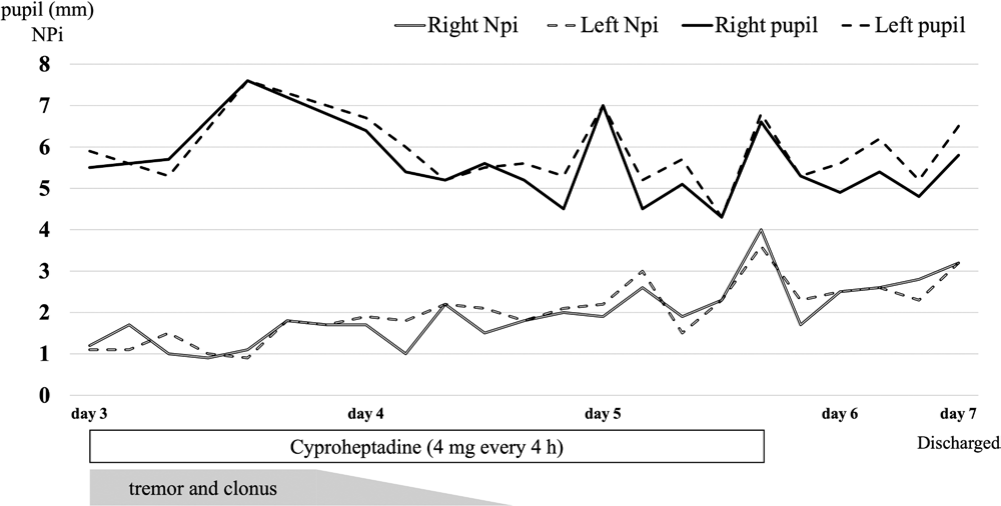
Clinical course of the patient. The NPi is noted with a double line and the pupillometer with a single line. In addition, the right eye is noted with a solid line and the left eye with a dotted line. On d 4, the tremor and clonus disappeared. There was a gradual improvement trend for NPi, but pupil diameter remained mostly unchanged. NPi = neurological pupil index.

On day 3, he was diagnosed with serotonin syndrome. Bacterial cultures were collected to evaluate the cause of fever; however, no significant bacteria were detected. Cyproheptadine (4 mg every 4 hours) was administered using nasogastric tube starting on day 3 and onwards. Pupillometers and NPi of both eyes were recorded using a digital pupillometer (NPi-200®︎, Neuroptics, Fig. 1) immediately before cyproheptadine administration. The strategy was to administer cyproheptadine if the NPi was below 3.0 in both eyes and terminate cyproheptadine when the NPi was above 3.0. On day 4, the tremor and clonus disappeared; however, the pupils still tended to dilate (5.5 mm/5.5 mm), and the NPi was low (1.7/1.9). On the same day, the patient was started on a solid diet, and administration using a nasogastric tube was switched to oral administration. NPi exceeded 3.0 in both eyes for the first time at 22:00 on day 5, and cyproheptadine administration was terminated. After the end of treatment, NPi decreased but began to increase again without any specific intervention. The pupil tended to be mildly dilated (5–6 mm in both eyes) during hospitalization. The patient desire to return home increased, and after a psychiatric consultation, he was discharged on day 7 (Fig. 2). The patient did not agree to a follow-up visit after discharge, which was terminated on the day of discharge.

## 3. Discussion

Serotonin, also known as 5-hydroxytryptamine, is a neurotransmitter. It is a monoamine regulating attention, behavior, thermoregulation, and mood stabilizer and provides moderate tension (e.g., antigravity muscle tone and sympathetic tone) for various activities in the waking state.^[5]^ Serotonin is present in only 2% of the central nervous system and is abundant in the body, mainly in the enterochromaffin cells of the small intestine, where it is involved in intestinal peristalsis.^[5]^ Postsynaptic stimulation of 5-HT1A and 5- HT2A receptors is said to induce serotonin syndrome, although it may be caused by a combination of drugs rather than a single receptor stimulation.^[6]^ Malignant syndromes are a different disease in that they are caused by dopamine antagonists. Here, the patient was presumed to have had a relatively severe condition owing to the overdose of 3 drugs: a serotonin agonist, serotonin-noradrenaline reuptake inhibitor, and serotonin-dopamine antagonist. Hence, 4 mg of cyproheptadine was administered repeatedly every 4 hours, and the NPi and pupil diameters were recorded on a digital pupillometer.

The diagnosis of serotonin syndrome was based on the clinical findings. However, there is no correlation between blood serotonin levels and clinical findings, and no laboratory tests can diagnose the serotonin syndrome.^[5]^ The Hunter criteria are the most accurate diagnostic criteria (84% sensitivity and 97% specificity compared with those of the gold standard for diagnosis by a medical toxicologist).^[2]^ In this case, tremors, spontaneous and induced clonus, and excitation were observed, and thus, the diagnosis was confirmed.

The serotonin syndrome treatment is focused on supportive care aimed at the discontinuation of the causative agent and stabilization of vital signs.^[1]^ Benzodiazepines may also be useful for agitation.^[1]^ Although most patients experience symptomatic improvement within 24 hours after discontinuation of the causative agent, it took 4 days for the tremor and clonus to improve in this case. Few more days were required for the improvement of the NPi and pupil diameter. This may be because the half-lives of mirtazapine, venlafaxine, and lurasidone are the longest, approximately 40, 11, and 40 hours, respectively,^[7–9]^ which may have caused their blood concentrations to remain high. In this case, blood concentrations of the 3 drugs could not be determined.

If supportive care does not improve the serotonin syndrome, cyproheptadine, a nonspecific serotonin receptor antagonist, may be considered.^[4]^ Cyproheptadine is available as a tablet or syrup and can be administered using a nasogastric tube. Our hospital uses a syrup formulation. The standard loading dose is 12 mg, followed by 2 mg every 2 hours, until a clinical response is seen.^[6]^ However, the exact method of administration remains unclear. In this case, because it was necessary to record NPi over time, doses were administered every 4 hours instead of every 2 hours. Cyproheptadine (68 mg) was administered; however, no considerable improvement was observed. A large variation in dosage exists for cyproheptadine, with certain individuals improving with 4 mg and others improving with 116 mg administered over a 7-day period.^[10]^ Serotonin syndrome has a good prognosis and may have a mild course, with or without cyproheptadine. As there is no conclusive evidence regarding the administration of cyproheptadine, further case reports may be able to highlight the optimal dosage.

In this case, pupillometry with a digital pupillometer and NPi were used as criteria to determine dysautonomic symptoms. Conventional measurement of pupil diameter using a penlight is problematic because there is considerable inter-examiner variation, and the light-to-light reflection is also subjectively evaluated as “normal” or “dull.” The digital pupillometer uses a 1000 lux infrared camera to measure the pupil diameter, quantitative counter-reflectance (i.e., the difference between baseline and post-stimulus pupil size and the rate of contraction from baseline values), and pupil contraction rate. In addition, the NPi was measured according to a proprietary algorithm and a value of <3.0 is considered an abnormal value.^[11]^ The usefulness of NPi has been reported in the field of intensive care and can be used for intracranial pressure monitoring and the evaluation of post-resuscitation encephalopathy after cardiopulmonary arrest.^[12]^ In this case, cyproheptadine was administered with a cutoff NPi of 3.0, but it took approximately 5 days for the NPi to exceed 3.0. On day 4, findings other than the pupil diameter showed improvement, thereby suggesting that the patient may have been hospitalized unnecessarily and that medication prescription with NPi as an indicator may have prolonged the length of hospital stay.

There are several limitations to this report. First, the initiation of cyproheptadine was delayed. Therefore, serotonin syndrome was likely to have developed from the time of admission, and had it been administered at that time, the results may have been different. Secondly, since NPi at the time of admission was not measured, we were unable compare the measurements.

## 4. Conclusion

We report the case of serotonin syndrome caused by an overdose that was treated with cyproheptadine. The NPi was recorded using a digital pupillometer. In this case, we did not observe a relationship between NPi and serotonin syndrome in a patient treated with cyproheptadine.

## Acknowledgments

We would like to thank Editage (www.editage.com) for English language editing.

## Author contributions

**Conceptualization:** Kazuki Sugaya.

**Investigation:** Kazuki Sugaya.

**Resources:** Tomotaka Misawa.

**Supervision:** Makoto Onodera, Ken Iseki.

**Writing – original draft:** Kazuki Sugaya.

**Writing – review & editing:** Kazuki Sugaya, Tomotaka Misawa, Makoto Onodera, Ken Iseki.
